# Size distribution of polycyclic aromatic hydrocarbons in space: an old new light on the 11.2/3.3 μm intensity ratio†

**DOI:** 10.1039/d2fd00180b

**Published:** 2023-02-02

**Authors:** Alexander K. Lemmens, Cameron J. Mackie, Alessandra Candian, Timothy M. J. Lee, Alexander G. G. M. Tielens, Anouk M. Rijs, Wybren Jan Buma

**Affiliations:** a Van 't Hoff Institute for Molecular Sciences, University of Amsterdam 1098 XH Amsterdam The Netherlands w.j.buma@uva.nl; b Radboud University, Institute for Molecules and Materials, FELIX Laboratory 6525 ED Nijmegen The Netherlands; c Kenneth S. Pitzer Center for Theoretical Chemistry, Department of Chemistry, University of California Berkeley California 94720 USA; d Chemical Sciences Division, Lawrence Berkeley National Laboratory Berkeley California 94720 USA; e Anton Pannekoek Institute for Astronomy, University of Amsterdam 1098 XH Amsterdam The Netherlands; f NASA Ames Research Center, Moffett Field California 94035-1000 USA; g Leiden Observatory, Leiden University 2333 CA Leiden The Netherlands; h Division of BioAnalytical Chemistry, AIMMS Amsterdam Institute of Molecular and Life Sciences, Faculty of Science, Vrije Universiteit Amsterdam De Boelelaan 1081 1081 HV Amsterdam The Netherlands

## Abstract

The intensity ratio of the 11.2/3.3 μm emission bands is considered to be a reliable tracer of the size distribution of polycyclic aromatic hydrocarbons (PAHs) in the interstellar medium (ISM). This paper describes the validation of the calculated intrinsic infrared (IR) spectra of PAHs that underlie the interpretation of the observed ratio. The comparison of harmonic calculations from the NASA Ames PAH IR spectroscopic database to gas-phase experimental absorption IR spectra reveals a consistent underestimation of the 11.2/3.3 μm intensity ratio by 34%. IR spectra based on higher level anharmonic calculations, on the other hand, are in very good agreement with the experiments. While there are indications that the 11.2/3.3 μm ratio increases systematically for PAHs in the relevant size range when using a larger basis set, it is unfortunately not yet possible to reliably calculate anharmonic spectra for large PAHs. Based on these considerations, we have adjusted the intrinsic ratio of these modes and incorporated this in an interstellar PAH emission model. This corrected model implies that typical PAH sizes in reflection nebulae such as NGC 7023 – previously inferred to be in the range of 50 to 70 carbon atoms per PAH are actually in the range of 40 to 55 carbon atoms. The higher limit of this range is close to the size of the C_60_ fullerene (also detected in reflection nebulae), which would be in line with the hypothesis that, under appropriate conditions, large PAHs are converted into the more stable fullerenes in the ISM.

## Abstract 2

Calculated infrared absorption intensities of PAHs that underlie the interpretation of infrared emission from the ISM are validated with experiments. An adjusted emission model implies that typical PAH sizes are smaller than previously estimated.

## Graphical abstract

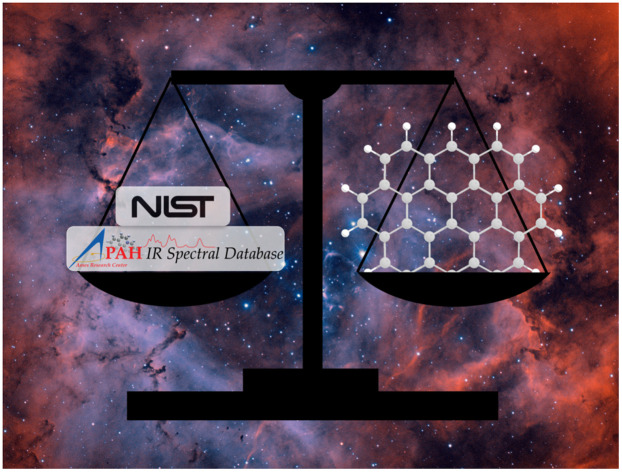

## 1. Introduction

The most widely accepted explanation for the emission of distinct infrared (IR) features, observed towards many different phases of the interstellar medium (ISM), is the PAH hypothesis.^1–3^ From different regions of the ISM a small, but clear variation of spectral profiles is observed, which is attributed to a variation in the population of PAHs in terms of size, structure and charge state.^4,5^ This variation is thought to be controlled by the local physical conditions.^6,7^ Since the introduction of the PAH hypothesis, much effort has gone into modeling the mechanisms of molecular IR emission aiming to place better constraints on the molecular inventory of the ISM and to learn how they are affected by their environment. Apart from the charge state, abundance and structure, one of the constraints that receives a lot of attention is the size of PAHs, mostly in terms of the number of carbon atoms.

The IR emission by isolated PAHs starts with the absorption of a UV photon. Because under the dilute conditions of the ISM the collisional cooling rate is much smaller than the spontaneous emission rate, most energy is lost by vibrational relaxation which is accompanied by the fluorescence of IR photons. The observed IR spectrum is an average of various PAH species relaxing and emitting IR photons *via* different relaxation cascades.^8^ Interstellar PAHs are exposed to a spectrum of UV photon energies, but in first approximation and because the UV photon energy does not vary a lot within objects the UV photon energy is often taken as a single value.^6^

Currently, the commonly accepted approach for deriving the size distribution of PAHs involves the intensity ratio of the 3.3 μm band to that of a band at a longer wavelength, typically the 11.2 μm band.^2,9–16^ The 3.3 μm peak corresponds to CH stretch vibrations and the relative intensity of this band was found to have the strongest (inverse) dependence on the number of carbon atoms within a PAH.^9^ The 11.2 μm peak is due to the CH out-of-plane (OOP) mode from solo Hs. The complete CH OOP region, which includes also the duo, trio and quartet H OOP modes, has been nicely decomposed and analyzed by Bauschlicher *et al.* and ranges from about 650–950 cm^−1^ (15.4 to 10.5 μm).^17^

The main reason behind the sensitivity of the 11.2/3.3 μm intensity ratio to molecular size is that smaller PAHs reach a higher temperature upon absorption of the same UV photon due to their smaller heat capacity, and therefore emit more photons at higher frequencies than larger PAHs.^4^ The heat capacity, and thereby the cascade starting temperature that results from the absorption of a particular UV photon, can be described in terms of isolated harmonic oscillators or alternatively be calculated using more general PAH properties (approximation by Stein and Dwek *et al.*).^18–20^ Additional advantages of using the 11.2/3.3 μm intensity ratio as a size tracer are that both bands originate mainly from CH bonds in neutral species and that they are spectrally well separated.^6^ Moreover, the influence of the number of hydrogen atoms or of the PAH molecular structure on the ratio is minimal.^12^

The NASA Ames PAH IR spectroscopic database (PAHdb) supports a number of analyses of the observed 11.2/3.3 μm ratio.^21^ It has been used to determine the PAH sizes in several astrophysical objects^12,22–25^ and quite recently Maragkoudakis *et al.* explored extensively how the 11.2/3.3 μm ratio relates to the PAH size distribution.^9^ The calculated 0 K absorption intensities (hereafter called intrinsic strengths) that underlie the emission models not only affect the emission ratio, but also the band profile. By artificially adjusting the intrinsic ratio it was found that a large 11.2/3.3 μm ratio results in a red-wing at the 11.2 μm feature which fades away at smaller ratios.^26^

The IR intensities in the PAHdb that are employed in the emission models are calculated at a relatively low level of theory (B3LYP/4-31G) and are based on the harmonic approximation. Because of the significant effect of intrinsic strengths on the outcome of the emission models in terms of ratio and peak profile, it is of key importance to validate these calculations with suitable experiments. Even more so because it was noticed already in the 90s by Langhoff and later by others that the intensity ratios derived at different levels of theory can vary significantly.^27–30^ First, the use of a small basis set results in an overprediction of CH stretch intensities by about 20% when compared to matrix-isolation absorption spectra.^27^ Secondly, it was recently shown that anharmonicity leading to phenomena such as combination bands and resonances has a dominant effect on the CH stretch region of the IR spectrum. These anharmonic contributions can carry significant intensity.^28,31^

Moreover, also from an experimental perspective it has been concluded that one needs to be cautious. Compared to the gas phase, the CH stretch intensity in IR absorption measurements in the solid phase is about a factor 3 lower.^32^ In matrix-isolation IR absorption measurements, on the other hand, the CH OOP intensity is about a factor 5 lower. Both experimental conditions will thus have a large effect on the 11.2/3.3 μm ratio.^32^

The aim of this study is to compare theory that is commonly used to interpret astronomical observations to experimental data in terms of intensity, utilizing direct absorption techniques for reliable intensities over a wide IR wavelength range. The theory at a (relatively) low vibrational temperature should be validated before extrapolating the models to describe the emission of highly excited PAHs and using these models to put constraints on the size distribution of PAHs. Gas-phase FTIR spectroscopy provides a good benchmark for oscillator strengths, because at the relatively low experimental temperatures there is no significant occupation of higher vibrational levels. In first approximation, a higher temperature will thus only lead to band broadening.^32,33^ Moreover, with FTIR spectroscopy the data are collected simultaneously over a wide spectral range. We will show that the present analysis leads to the conclusion that previously employed emission models need to be reconsidered, and, importantly, that the application of modified models predicts notably smaller PAH sizes than before.

## 2. Methods

**Fig. 1:**
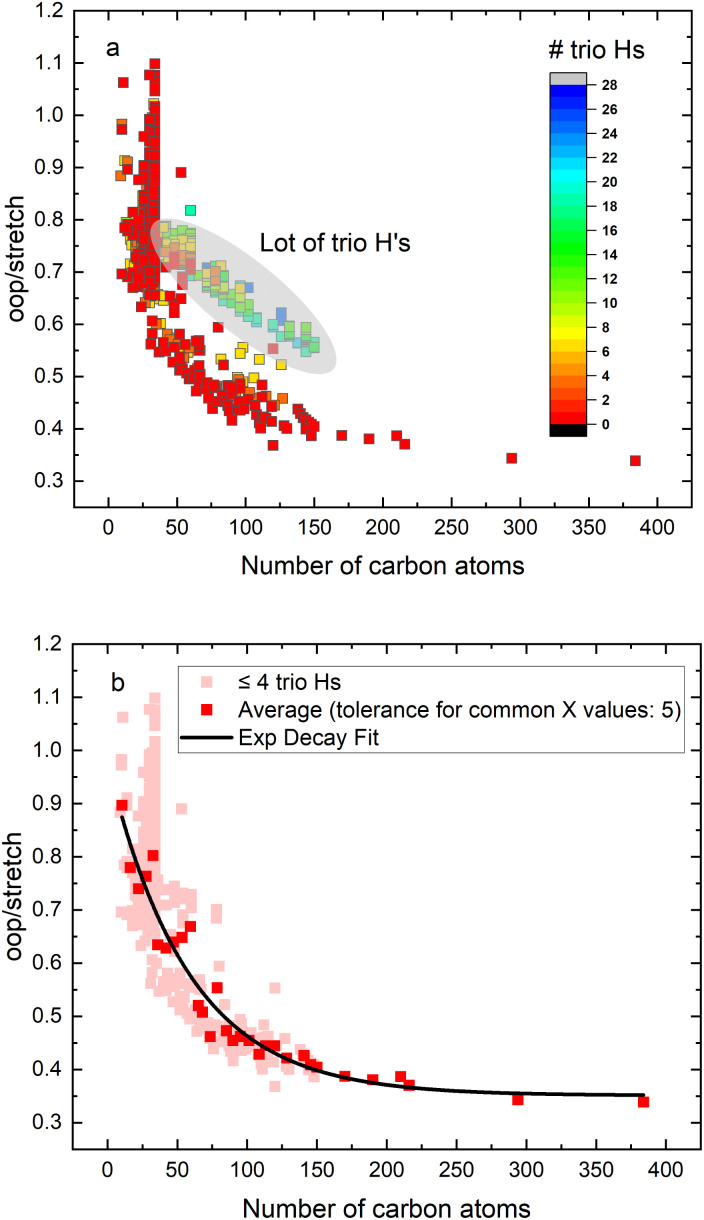
(a) The intrinsic oop/stretch intensity ratio of neutral PAHs in the PAHdb as a function of the number of carbon atoms. The color scale corresponds to the number of trio hydrogens in the PAH. (b) Same plot as (a), but leaving out PAHs that contain more than 4 trio hydrogens (light red). The dark red symbols represent the average for fixed sizes (size tolerance is 5 carbon atoms). The black line is an exponential decay fit to the latter.

We compared (relative) intensities from the IR spectra of PAHs from four different experimental and theoretical sources, namely the NASA Ames PAH IR spectroscopic database (PAHdb, version 3.20),^21^ anharmonic calculations extracted from the work by Mackie *et al.*^34^ or performed here using the Gaussian 16 software package, experimental gas-phase FT-IR absorption spectra from the NIST database^35^ and experimental matrix isolation IR spectra extracted from the PAHdb.^36^ To study the relation between molecular size and the intrinsic 3.3/11.2 μm ratio, we extracted theoretical PAH spectra from the PAHdb of neutral PAHs that do not contain any oxygen or nitrogen atoms (Fig. 1). The intensity is integrated between 650–950 cm^−1^ (15.4 and 10.5 μm) and will be indicated in the following as ‘oop’. The integrated range for the CH stretch region, indicated in the following as ‘stretch’, spans from 2750–3250 cm^−1^ (3.6–3.1 μm). For the ratio in emission (Fig. 3) we selected neutral PAHs that contain more than one hydrogen atom, no aliphatic groups and did not include Fe, Si, Mg, N, or O substituted species. Finally, we only considered PAHs with solo (more than 4) and duo hydrogens, as they are generally more compact and thus more stable. One can reasonably expect that this restriction will only affect to a minor extent the conclusions as the structure of the PAH should not have a large influence on the oop/stretch emission ratio.^27,32^ Calculations referred to as anharmonic are either described previously and extracted from ref. 34, or they have been performed at the anharmonic^37,38^ B3LYP^39^/N07D^40,41^ level of theory using Gaussian 16 (ref. 42) as indicated.

## 3. Results and discussion

### 3.1 Theory and gas-phase experiments

In order to investigate the intrinsic intensity ratio as a function of PAH size, Fig. 1a shows the band strength ratio extracted from the (harmonically calculated) PAH database for the complete available size range. Fig. 1a shows that this ratio varies by a factor of about 3 between the smallest and largest PAHs considered here. Interestingly, a large variation of the band strength ratio is associated with small PAHs containing up to about 35 carbon atoms, which results in a larger uncertainty in the calculated emission ratio over this size range. The color scale of Fig. 1a corresponds to the number of trio hydrogens in the PAH. A clear separation of PAHs that contain more than 4 trio hydrogens is visible from PAHs with 4 or less trio hydrogens. The presence of trio hydrogens increases the oop/stretch ratio by suppressing the CH stretch band intensity.

In the most stable PAH species, one does not expect many trio hydrogens as they are in general associated with an irregular shape rather than a compact one.^43^ To further investigate the relation between the intrinsic oop/stretch ratio and PAH size, PAHs with more than 4 trio hydrogens have therefore been omitted in Fig. 1b. Moreover, to remove the bias towards the more numerous smaller PAHs an average has been taken for fixed sizes (tolerance 5 carbon atoms). Quite interestingly, we then find that the oop/stretch ratio and the number of carbon atoms in a PAH exhibit a clear drop with size that converges to an intrinsic oop/stretch ratio of 0.35 for large PAHs. This drop can be well represented by an exponential function given by
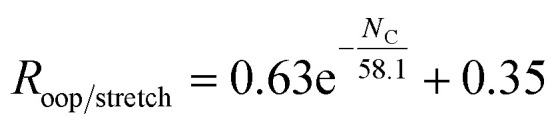
With *R*_oop/stretch_ the intrinsic oop/stretch intensity ratio and *N*_C_ the number of carbon atoms.

**Fig. 2:**
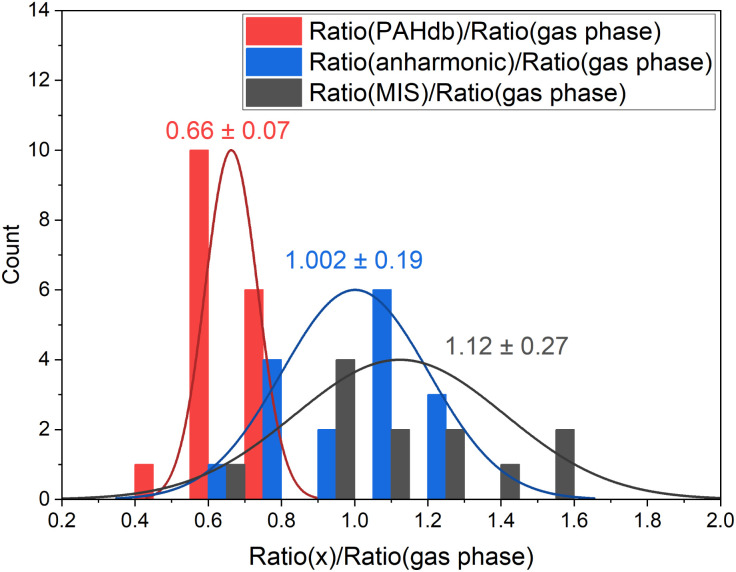
Histogram of the oop/stretch intensity ratio determined using different theoretical and experimental methods divided by the gas-phase direct absorption measurements. The bin size is 0.15. The intensities were integrated between 650–950 cm^−1^ (15.4–10.5 μm) and 2750–3250 cm^−1^ (3.6–3.1 μm). The PAH species that are taken into consideration are listed in the ESI.† The mean and deviation of the distributions are indicated in the plot.

The large number of theoretical IR spectra in the PAHdb have provided extensive insight into the interstellar PAH population and spectroscopic properties of PAHs. However, considering the intensities, the harmonic, relatively low-level calculations are known to consistently overestimate the CH stretch band intensity.^29^ To corroborate these findings, in Fig. 2 we compare the calculated ratio from the PAHdb with the ratio determined from gas-phase direct absorption measurements from the NIST database.^35^ As discussed in the introduction, anharmonic calculations lead to a significant improvement in predicting IR spectra. The improvement mainly lies in the addition of combination bands and resonances that carry a significant amount of intensity, especially in the CH stretch region. Fig. 2 thus reports on a comparison in terms of intensities resulting from such calculations. Finally, a comparison is made between the gas-phase absorption measurements to cold, matrix isolation absorption measurements.

The oop/stretch intensity ratio determined using the theoretical spectra of the PAHdb is smaller than the experimental gas-phase ratio by a factor of 0.66. This corroborates the previous findings that the CH stretch intensities are overestimated, although this overestimation appears to be even slightly larger than reported previously.^27^ Interestingly, the ratio is quite consistently off, which supports the approach to scale the band strengths computed at the B3LYP/4-31G level to obtain more accurate ratios. This approach was used before to obtain band strengths for methylated PAHs that were as accurate as those calculated at far more expensive levels (MP2/6-311+G(3df,3pd)).^44^ We compare the DFT calculated ratios for the coronene family for different basis sets and find that upon increasing the basis set the oop/stretch intensity ratio increases (see Table S2†).

The ratios that result from anharmonic calculations show on average a remarkably good agreement with the gas-phase absorption experiments in terms of accuracy. It should be noted that a larger basis set (N07D, which is a modification of 6-31G*) is used in these anharmonic calculations than has been used for the PAHdb and such a larger basis set is expected to lead to a more accurate ratio, mainly resulting from a smaller stretch intensity.^29^ Nevertheless, examination of the effects of the inclusion of anharmonicity on the ratio is of great interest as a significant amount of intensity is involved in the combination bands and resonances. The ratio determined from matrix isolation spectroscopy measurements shows the largest spread with respect to gas-phase absorption experiments. The comparison of the ratios of individual PAHs can be found in Fig. S1 in the ESI.†

### 3.2 Correcting for systematic deviation

**Fig. 3:**
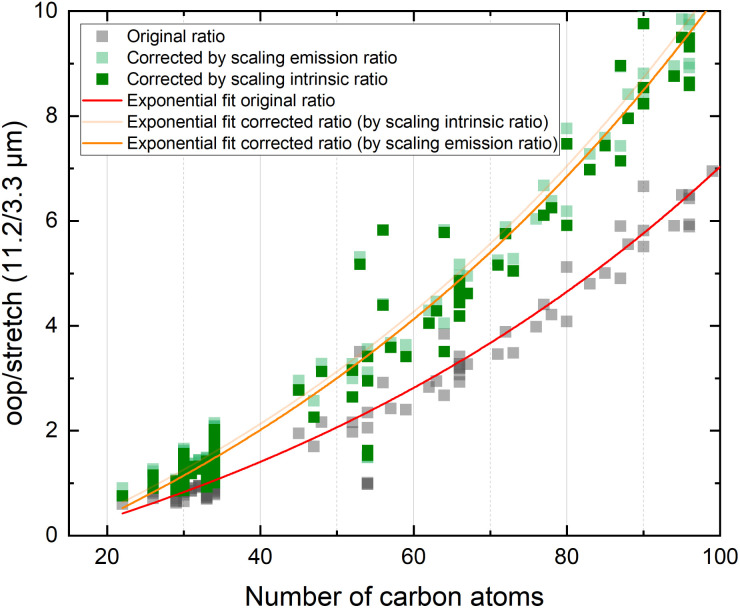
Grey squares indicate the oop/stretch intensity ratio as a function of the number of carbon atoms determined using the calculated intrinsic band strengths and a full temperature cascade emission model from the PAHdb on a large selection of PAHs (for details, see the Methods section). The green squares are scaled by a factor of 1/0.66 (see text) to correct for the systematic underestimation of the intrinsic oop/stretch intensity ratio by low-level calculations. Intensities were integrated between 830–985 cm^−1^ (12.05–10.15 μm) and 2950–3115 cm^−1^ (3.4–3.2 μm).

In view of the above observations, it becomes quite interesting to determine how the underestimation of the oop/stretch intensity ratio affects conclusions on the PAH size distributions. To this purpose, we used the harmonically calculated intrinsic band strengths and applied a full temperature cascade emission model from the PAHdb at 6.5 eV on a large selection of PAHs (for details, see the Methods section). The results are shown in Fig. 3 (grey squares). In order to correct for the underestimation of the intrinsic oop/stretch intensity ratio by low-level calculations, a coarse modification has been made by scaling the emission ratio with the factor by which the low-level theory calculations underestimate the intrinsic ratio (1/0.66, light-green squares in Fig. 3). Such an approach was used before for methylated PAHs, where the band strengths of inexpensive calculations were scaled to match the more expensive predictions.^44^ This figure also displays the results of a more accurate approach in which the intrinsic ratio is scaled and afterwards the full temperature cascade emission model is applied (dark-green squares). Aiming to facilitate a readout of these plots and serving as a guide to the eye, the data sets have been fitted with an exponential function.

**Fig. 4:**
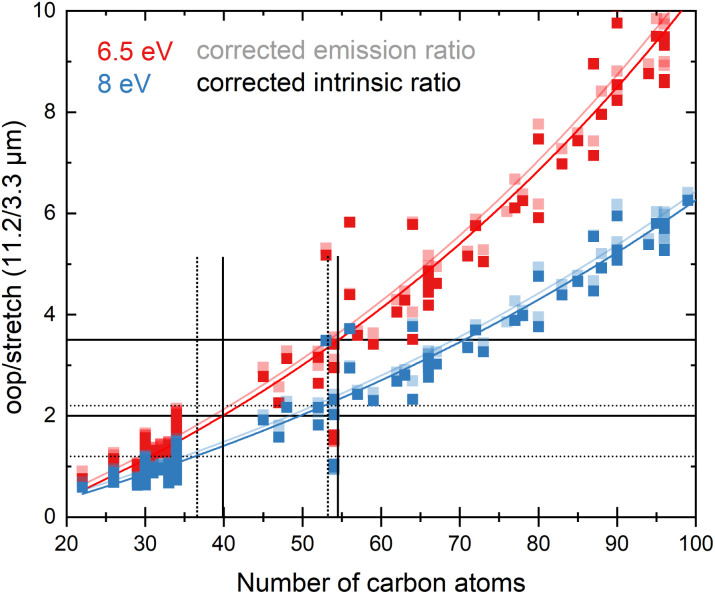
The oop/stretch intensity ratio as a function of the number of carbon atoms determined using the calculated band strengths and a full temperature cascade emission model from the PAHdb on a large selection of PAHs. The transparent squares correspond to the ratio scaled after applying the emission model, the opaque squares to scaling the intrinsic strengths before applying the emission model, both cases aiming to correct for the systematic underestimation of the intrinsic oop/stretch intensity ratio by low-level calculations. The solid and dotted black horizontal lines are examples of lower and higher limits of the observed intensity ratio in NGC 7023 and the Orion Bar, respectively.^12,24^ The exponential fits indicated with the colored solid lines are phenomenological and meant to facilitate readout (see Table S1† for fit parameters).

Since the average absorbed UV photon energy varies between astronomical objects, we have calculated the emission ratio as a function of molecular size for cascade starting energies of 6.5 and 8 eV, these energies being representative for the average photon energy of a reflection nebula such as NGC 7023 (ref. 12) and for a Photon Dominated Region such as the Orion Bar,^24^ respectively. We assume one-photon absorption processes as cooling rates are high compared to absorption rates in the objects studied here.^6^ Multiphoton events would result in a decrease of the oop/stretch ratio. The results of these calculations are shown in Fig. 4 with the ratio of correcting both before and after applying the emission model plotted as transparent and opaque color, respectively. While correcting the intensity ratio before applying the emission model is most appropriate, we also include the results derived by applying the correction factor after applying the emission models as a convenient way to interpret the results of the current PAHdb. An exponential function to facilitate readout has been fitted to all plots and is provided in the ESI.†

With both the ‘original’ model and the corrected model, the 11.2/3.3 μm intensity ratio can now be translated into a PAH size distribution. The two horizontal solid black lines in Fig. 4 are typical lower and higher limits of observed intensity ratios in NGC 7023.^12^ The two horizontal dotted black lines indicate lower and higher limits of the intensity ratios in the Orion Bar.^24^ For NGC 7023, using the ‘original’ model and taking the indicated upper and lower observed ratio limits, one would conclude that typical PAH sizes are in the 50–70 carbon atoms range. Importantly, however, we find that when using the corrected model which takes the systematic underestimation of the intensity ratio into account, this range is reduced to 40–55 carbon atoms. For the Orion Bar region, the PAH size range is determined to be between 37–53 carbon atoms.

The results of the present model are quite remarkable but need to be substantiated further with more extensive analyses that incorporate accurate high-level anharmonic calculations on PAHs with a wide range of sizes in the emission model. Such analyses are now indeed high on our priority list. However, as for larger PAHs these calculations are not available yet, this model awaits completion. Nevertheless, our results do indicate that the underestimation of the oop/stretch intensity ratio by low-level calculations translates into a significant overestimation of the PAH size distribution. Equally important to notice is that our results are still in line with the hypothesis that an interconversion between large PAHs and the even more stable C_60_ fullerene takes place at a certain level of the UV radiation field.^7^ From the higher limit of about 55 carbon atoms that our results imply, it appears that PAHs containing more than 60 carbon atoms should indeed be readily converted into fullerene species.

## 4. Conclusions

By comparing calculations from the NASA Ames PAH IR spectroscopic database (PAHdb) and state-of-the-art anharmonic calculations with gas-phase direct absorption IR spectra from the NIST chemistry webbook, this study validates the predicted intrinsic oop/stretch intensity ratio of PAHs. Currently, this ratio is recognized as the most reliable tracer for determining the size distribution of PAHs in the interstellar medium. Our studies show that the relatively low-level calculations that have been employed so far to interpret astronomical observations consistently underestimate the intrinsic intensity ratio by about 34%. Predictions made with a larger basis-set and taking anharmonic effects such as combination bands and resonances into account are, in contrast, in good agreement with the experiment. Unfortunately, the high-level anharmonic calculations are not available yet for large PAHs. Nevertheless, the consistency of the underestimation of the intensity ratio allows for making an *ad hoc*, coarse correction to the emission model based on the PAHdb. Such a corrected ratio–PAH size relation suggests that the size distribution, which up till now has been taken to be in the 50–70 carbon atoms range, could very well need to be adjusted to the 40–55 range. It is quite satisfying to notice that the higher limit of the PAH size conforms to the hypothesis that an interconversion takes place between larger sized PAHs and fullerenes in the interstellar medium.

## Conflicts of interest

There are no conflicts to declare.

## Supplementary Material

FD-245-D2FD00180B-s001

## References

[cit1] Tielens A. G. G. M. (2008). Annu. Rev. Astron. Astrophys..

[cit2] Allamandola L. J., Tielens A. G. G. M., Barker J. R. (1989). Astrophys. J., Suppl. Ser..

[cit3] Leger A., Puget J. L. (1984). Astron. Astrophys..

[cit4] Peeters E., Mackie C., Candian A., Tielens A. G. G. M. (2021). Acc. Chem. Res..

[cit5] Galliano F., Madden S. C., Tielens A. G. G. M., Peeters E., Jones A. (2008). Astrophys. J..

[cit6] Tielens A. G. G. M. , Molecular Astrophysics, Cambridge University Press, Cambridge, 2021

[cit7] Berné O., Tielens A. G. G. M. (2012). Proc. Natl. Acad. Sci. U. S. A..

[cit8] Mackie C. J., Chen T., Candian A., Lee T. J., Tielens A. G. G. M. (2018). J. Chem. Phys..

[cit9] Maragkoudakis A., Peeters E., Ricca A. (2020). Mon. Not. R. Astron. Soc..

[cit10] Schutte W. A., Tielens A. G. G. M., Allamandola L. J. (1993). Astrophys. J..

[cit11] Ricca A., Bauschlicher C. W., Boersma C., Tielens A. G. G. M., Allamandola L. J. (2012). Astrophys. J..

[cit12] Croiset B. A., Candian A., Berné O., Tielens A. G. G. M. (2016). Astron. Astrophys..

[cit13] Jourdain de Muizon M., Cox P., Lequeux J. (1990). Astron. Astrophys., Suppl. Ser..

[cit14] Mori T. I., Sakon I., Onaka T., Kaneda H., Umehata H., Ohsawa R. (2012). Astrophys. J..

[cit15] Pech C., Joblin C., Boissel P. (2002). Astron. Astrophys..

[cit16] Andrews H., Boersma C., Werner M. W., Livingston J., Allamandola L. J., Tielens A. G. G. M. (2015). Astrophys. J..

[cit17] Bauschlicher C. W., Peeters E., Allamandola L. J. (2009). Astrophys. J..

[cit18] Stein S. E., Spencer J. N., Seigart J. R., Brown E., Sensing R. L., Stein S. E. (1978). J. Phys. Chem..

[cit19] Dwek E., Arendt R. G., Fixsen D. J., Sodroski T. J., Odegard N., Weiland J. L., Reach W. T., Hauser M. G., Kelsall T., Moseley S. H., Silverberg R. F., Shafer R. A., Ballester J., Bazell D., Isaacman R. (1997). Astrophys. J..

[cit20] Bauschlicher C. W., Boersma C., Ricca A., Mattioda A. L., Cami J., Peeters E., Armas D., Saborido G. P., Hudgins D. M., Allamandola L. J. (2011). Astrophys. J., Suppl. Ser..

[cit21] Bauschlicher C. W., Ricca A., Boersma C., Allamandola L. J. (2018). Astrophys. J., Suppl. Ser..

[cit22] Boersma C., Bregman J., Allamandola L. J. (2015). Astrophys. J..

[cit23] Draine B. T., Li A., Hensley B. S., Hunt L. K., Sandstrom K., Smith J.-D. T. (2021). Astrophys. J..

[cit24] Knight C., Peeters E., Tielens A. G. G. M. (2022). Mon. Not. R. Astron. Soc..

[cit25] Knight C., Peeters E., Stock D. J., Vacca W. D., Tielens A. G. G. M. (2021). Astrophys. J..

[cit26] Mackie C. J., Candian A., Lee T. J., Tielens A. G. G. M. (2021). Theor. Chem. Acc..

[cit27] Langhoff S. R. (1996). J. Phys. Chem..

[cit28] Mackie C. J., Candian A., Huang X., Maltseva E., Petrignani A., Oomens J., Buma W. J., Lee T. J., Tielens A. G. G. M. (2015). J. Chem. Phys..

[cit29] Geindre H., Allouche A. R., Peláez D. (2021). J. Comput. Chem..

[cit30] Mackie C. J., Candian A., Lee T. J., Tielens A. G. G. M. (2022). J. Phys. Chem. A.

[cit31] Lemmens A. K., Rap D. B., Thunnissen J. M. M., Mackie C. J., Candian A., Tielens A. G. G. M., Rijs A. M., Buma W. J. (2019). Astron. Astrophys..

[cit32] Joblin C., D'Hendecourt L., Leger A., Defourneau D. (1994). Astron. Astrophys..

[cit33] Joblin C., Boissel P., Léger A., D'Hendecourt L., Défourneau D. (1995). Astron. Astrophys..

[cit34] Mackie C. J. , The Anharmonic Infrared Spectra of Polycyclic Aromatic Hydrocarbons, 2018 10.1063/1.5038725 30292208

[cit35] NIST Mass Spectrometry Data Center , Wallace W. E., director, in NIST Chemistry WebBook, NIST Standard Reference Database Number 69, ed. P. J. Linstrom and W. G. Mallard, National Institute of Standards and Technology, Gaithersburg, MD, Infrared Spectra, accessed Jan 2022

[cit36] Mattioda A. L., Hudgins D. M., Boersma C., Ricca A., Peeters E., Cami J., Sánchez de Armas F., Puerta Saborido G., Bauschlicher C. W., Allamandola L. J. (2020). Astrophys. J..

[cit37] Barone V. (2005). J. Chem. Phys..

[cit38] Barone V., Biczysko M., Bloino J. (2014). Phys. Chem. Chem. Phys..

[cit39] Becke A. D. (1993). J. Chem. Phys..

[cit40] Barone V., Cimino P., Stendardo E. (2008). J. Chem. Theory Comput..

[cit41] Mackie C. J., Candian A., Huang X., Maltseva E., Petrignani A., Oomens J., Buma W. J., Lee T. J., Tielens A. G. G. M. (2018). Phys. Chem. Chem. Phys..

[cit42] Frisch M. J. , Trucks G. W., Schlegel H. B., Scuseria G. E., Robb M. A., Cheeseman J. R., Scalmani G., Barone V., Petersson G. A., Nakatsuji H., Li X., Caricato M., Marenich A. V., Bloino J., Janesko B. G., Gomperts R., Mennucci B., Hratchian H. P., Ortiz J. V., Izmaylov A. F., Sonnenberg J. L., Williams-Young D., Ding F., Lipparini F., Egidi F., Goings J., Peng B., Petrone A., Henderson T., Ranasinghe D., Zakrzewski V. G., Gao J., Rega N., Zheng G., Liang W., Hada M., Ehara M., Toyota K., Fukuda R., Hasegawa J., Ishida M., Nakajima T., Honda Y., Kitao O., Nakai H., Vreven T., Throssell K., Montgomery Jr J. A., Peralta J. E., Ogliaro F., Bearpark M. J., Heyd J. J., Brothers E. N., Kudin K. N., Staroverov V. N., Keith T. A., Kobayashi R., Normand J., Raghavachari K., Rendell A. P., Burant J. C., Iyengar S. S., Tomasi J., Cossi M., Millam J. M., Klene M., Adamo C., Cammi R., Ochterski J. W., Martin R. L., Morokuma K., Farkas O., Foresman J. B. and Fox D. J., Gaussian 16, Revision A.03, Gaussian, Inc., Wallingford CT, 2016

[cit43] Bouwman J., Castellanos P., Bulak M., Terwisscha van Scheltinga J., Cami J., Linnartz H., Tielens A. G. G. M. (2019). Astron. Astrophys..

[cit44] Yang X. J., Li A., Glaser R., Zhong J. X. (2017). Astrophys. J..

