# Supplementary material for: Size distribution of polycyclic aromatic hydrocarbons in space: an old new light on the 11.2/3.3 μm intensity ratio
Source: Faraday Discuss. 2023 Feb 2;245:380–90. doi: 10.1039/d2fd00180b (PMC10510036; doi:10.1039/d2fd00180b)
Supplement: FD-245-D2FD00180B-s001 [file FD-245-D2FD00180B-s001.pdf]

Electronic Supplementary Material (ESI) for Faraday Discussions.
This journal is © The Royal Society of Chemistry 2023

Supporting Information for

Size distribution of polycyclic aromatic hydrocarbons in space: an old new light on the 11.2/3.3 µm intensity ratio

Alexander K. Lemmens[a,b], Cameron J. Mackie[c,d], Alessandra Candian[a,e], Timothy J. Lee[f], Alexander G. G. M. Tielens[g], Anouk M. Rijs[h], Wybren Jan Buma*[a,b]

[a] *van 't Hoff Institute for Molecular Sciences, University of Amsterdam, 1098 XH Amsterdam, The Netherlands. w.j.buma@uva.nl.*
[b] *Radboud University, Institute for Molecules and Materials, FELIX Laboratory, 6525 ED Nijmegen, The Netherlands.*
[c] *Kenneth S. Pitzer Center for Theoretical Chemistry, Department of Chemistry, University of California, Berkeley, California 94720, United States.*
[d] *Chemical Sciences Division, Lawrence Berkeley National Laboratory, Berkeley, California 94720, United States.*
[e] *Anton Pannekoek Institute for Astronomy, University of Amsterdam, 1098 XH Amsterdam, The Netherlands.*
[f] *NASA Ames Research Center, Moffett Field, California 94035-1000, United States.*
[g] *Leiden Observatory, Leiden University, 2333 CA Leiden, The Netherlands.*
[h] *Division of BioAnalytical Chemistry, AIMMS Amsterdam Institute of Molecular and Life Sciences, Faculty of Science, Vrije Universiteit Amsterdam, De Boelelaan 1081, 1081 HV Amsterdam, The Netherlands.*

Table S1: Fit parameters (y = A1*exp(-x/t1) + y0) corresponding to figure 4.

**6.5 eV** scaled before model		scaled after model		**8 eV** scaled before model		scaled after model	
y0	-4.97 ± 0.44	y0	-5.25 ± 0.52	y0	-3.91 ± 0.40	y0	-4.15 ± 0.48
A1	4.20 ± 0.33	A1	4.37 ± 0.41	A1	3.50 ± 0.33	A1	3.66 ± 0.40
t1	76.14 ± 2.68	t1	78.47 ± 3.29	t1	92.29 ± 4.34	t1	95.57 ± 5.30

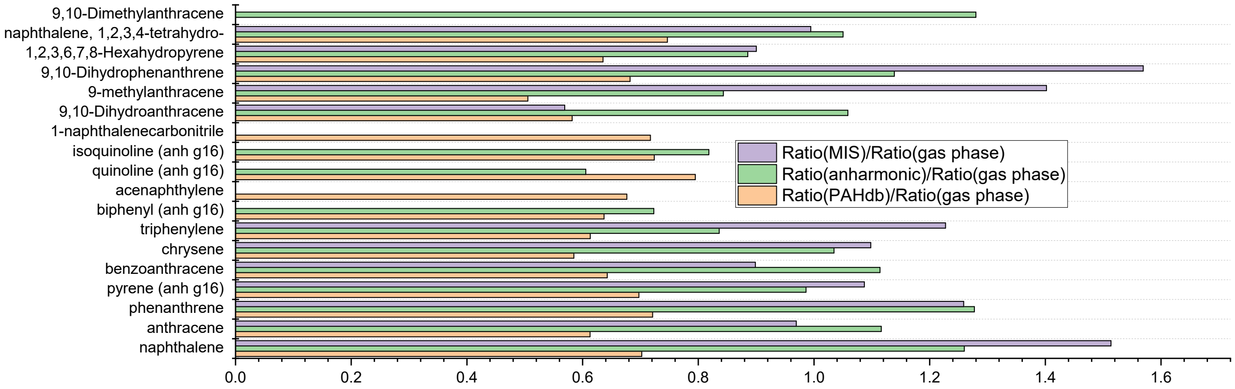

Figure S1: The 11.2/3.3 µm intensity ratio for different PAH species determined using various theoretical and experimental methods divided by the gas-phase direct absorption measurements. The intensities were integrated between 15.4-10.5 µm (650-950 cm^{-1}) and 3.6-3.1 µm (2750-3250 cm^{-1}).

Table S2: The 11.2/3.3 µm intensity ratio for different species of the coronene family determined using various basis sets. The intensities were integrated between 15.4-10.5 µm (650-950 cm^{-1}) and 3.6-3.1 µm (2750-3250 cm^{-1}). Calculations have been performed using Gaussian16 employing the B3LYP functional. For some of the reported calculations, in particular for larger species with larger basis sets, imaginary frequencies are observed. These imaginary modes correspond to out-of-plane vibrational modes and are indicated with an asterisk.

species\basis set	4-31G	6-31G(d,p)	6-311+G**	N07D
coronene ($C_{24}H_{12}$)	0.63	0.65	0.90	0.97
circumcoronene ($C_{54}H_{18}$)	0.53	0.52	0.65	0.80*
circumcircumcoronene ($C_{96}H_{24}$)	0.44	0.44	0.53	0.68*
circumcircumcircumcoronene ($C_{150}H_{30}$)	0.40	0.42*	0.48*	
circumcircumcircumcircumcoronene ($C_{216}H_{36}$)	0.37	0.38*		
